# Growth Factors in Proliferative Diabetic Retinopathy

**DOI:** 10.1155/EDR.2003.287

**Published:** 2003

**Authors:** Zia Ali Khan, Subrata Chakrabarti

**Affiliations:** 1 Department of Pathology University of Western Ontario Dental Sciences Building London Ontario N6A 5C1 Canada; 2 Department of Microbiology and Immunology University of Western Ontario London Ontario Canada

## Abstract

Many growth factors are implicated in the pathogenesis of proliferative diabetic retinopathy. Alteration of growth factors and their receptors in diabetes has been shown in both experimental and clinical studies. Sustained hyperglycemia resulting from long-standing diabetes leads to several biochemical abnormalities that consequently result in retinal hypoxia. Retinal oxygenation state regulates various growth factors that promote angiogenesis in order to meet the oxygen demands of the tissue. However, unregulated expression of these growth factors and induction of complex cascades leading to augmentation of other proangiogenic factors, which may not be regulated by tissue oxygenation, leads to uncontrolled retinal neovascularization and blindness in diabetic patients.


*Experimental Diab. Res.*, 4:287–301, 2003
Copyright © Taylor and Francis Inc.
ISSN: 1543-8600 print / 1543-8619 online
DOI: 10.1080/15438600390249727

### Growth Factors in Proliferative Diabetic Retinopathy

**Zia Ali Khan[1] and Subrata Chakrabarti[1,2]**

*Departments of [1]Pathology and [2]Microbiology and Immunology, University of Western Ontario, London, Ontario, Canada*

**Many growth factors are implicated in the pathogenesis of proliferative diabetic retinopathy. Alteration of growth factors and their receptors in diabetes has been shown in both experimental and clinical studies. Sustained hyperglycemia resulting from long-standing diabetes leads to several biochemical abnormalities that consequently result in retinal hypoxia. Retinal oxygenation state regulates various growth factors that promote angiogenesis in order to meet the oxygen demands of the tissue. However, unregulated expression of these growth factors and induction of complex cascades leading to augmentation of other proangiogenic factors, which may not be regulated by tissue oxygenation, leads to uncontrolled retinal neovascularization and blindness in diabetic patients.**

### INTRODUCTION

Diabetic retinopathy is a vascular complication of both type I and type II diabetes. Nearly all people with type I and more than half with type II diabetes develop complications involving the retina (Fong et al., 2003). Clinically, diabetic retinopathy can be classified as background retinopathy (BDR), preproliferative retinopathy (PPDR), and proliferative retinopathy (PDR) (Alder et al., 1997; Davis, 1992; Hudson, 1996; Neely et al., 1998). BDR is the earliest stage, which is characterized by capillary basement membrane thickening, pericyte loss, microaneurysms, increased permeability, exudate deposits, and retinal microinfarcts. Preproliferative retinopathy, on the other hand, is an advanced stage of retinopathy that subsequently leads to the proliferative stage. Progression to the proliferative stage results in neovascularization and accompanying hemorrhages.

Retinal changes in diabetes are thought to be initiated by sustained hyperglycemia leading to biochemical anomalies and alterations of various vasoactive factors and growth factors (Brownlee, 2001; Engerman et al., 1985; Diabetes Control and Complications Trial Research Group, 1993; King and Brownlee, 1996; Sheetz and King, 2002). Biochemical changes that are believed to alter the structural and functional properties of the retina include nonenzymatic glycation (Bierhaus et al., 1998; Brownlee et al., 1988; Kern and Engerman, 2001; Vlassara, 2001; Vlassara, 1997; Takagi et al., 1995), augmented polyol pathway and pseudohypoxia due to redox imbalance (Costantino et al., 1999; Engerman et al., 1993; Greene et al., 1987; Ido et al., 1997; Pugliese et al., 1991; Williamson et al., 1993), oxidative stress (Brownlee, 2001; Pugliese et al., 1991; Sheetz and King, 2002), and activation of protein kinase C (PKC) (Derubertis and Craven, 1994; Ishii et al., 1996; Ishizuka et al., 1989; Johannes et al., 1994; Koya and King, 1998; Lynch et al., 1990; Okumura et al., 1991; Williams et al., 1997; Xia et al., 1994). In addition, there is also alteration in the expression of several growth factors, vasoactive factors, and their respective receptors. Early in the disease course, these biochemical anomalies lead to alteration of retinal blood flow, in part because of impaired nitric oxide activity and up-regulated endothelin expression (Brownlee, 2001; Sheetz and King, 2002). With progression of the disease, vascular cells lose intercellular tight junctions and become susceptible to degeneration, the

Received 13 February 2003; accepted 12 April 2003.

The authors acknowledge grant support from Canadian Diabetes Association, in honor of Margaret Francis, and from Lawson Health Research Institute.

Address correspondence to Dr. Subrata Chakrabarti, Department of Pathology, Dental Sciences Building, University of Western Ontario, London, Ontario N6A 5C1, Canada. E-mail: schakrab@uwo.ca aftermath being increased permeability. Elaboration of growth factors such as vascular endothelial growth factor (VEGF) and endothelins, stimulated by hyperglycemia and cellular degeneration, lead to further increased permeability and increased extracellular matrix (ECM) protein deposition. A schematic outline of these events and their possible interactions are presented in Figure 1.

Angiogenesis is a complex but normal process under various physiological conditions. However, when unregulated, it may be a principle mechanism of tissue damage in specific pathological conditions, as is the case in PDR. Angiogenesis may be the most devastating outcome of growth factor alterations in PDR. Retinal neovascularization is believed to be the consequence of hypoxia due to retinal capillary closure. This idea is supported by studies that have shown concurrent occurrence of hypoxia and neovascularization (Patz, 1982; Shimizu et al., 1981). This hypoxia-induced state is believed to cause functional alterations in various cell types, including glial cells, endothelial cells, retinal pigment epithelial cells, vascular smooth muscle cells, fibroblasts, and inflammatory cells. However, the primary target of glucose-induced dysfunction is endothelial cells (Chakir and Plante, 1996; De Vriese et al., 2001; Hink et al., 2001; King et al., 1994) and they are therefore most susceptible to proliferating signals.

Numerous growth factors have been implicated in the pathogenesis of PDR. Growth factor alterations are believed to be important in both early and late stages of diabetic retinopathy. Early events in the disease course, dictated in part by growth factor alterations, include basement membrane thickening, a hallmark of chronic diabetic complications. Pericyte loss and endothelial cell damage are two factors that may further aggravate a growth factor–mediated proliferative response, resulting in increased ECM deposition and basement membrane thickening (Williamson and Kilo, 1983). In addition, various growth factors are involved in later stages of diabetic retinopathy, leading to proliferation and migration of endothelial cells and subsequent neovascularization (Casey and Li, 1997; D'Amore, 1994; Grant et al., 1987; Gross et al., 1983; Sholley et al., 1984). Several of these growth factors are also believed to participate in the balance of ECM protein expression and degradation, a phenomenon integral to angiogenesis (Hink et al., 2001; Ishii et al., 1996; Patz, 1982; Shimizu et al., 1981; Williams et al., 1997). Proteinases called plasminogen activators (PAs) carry out the conversion of plasminogen to plasmin, which plays an important role in the proteolysis of the ECM proteins. Other proteases, matrix metalloproteinases (MMPs), also degrade various ECM proteins. Naturally occurring inhibitors of PAs and MMPs, such as plasminogen activator inhibitors (PAIs) and tissue inhibitor of matrix metalloproteinases (TIMPs) are, in addition to PAs and MMPs, believed to be regulated by various growth factors (Casey and Li, 1997; D'Amore, 1994; Grant et al., 1987; Gross et al., 1983; Sholley et al., 1984).

The involvement of growth factors in PDR is supported by studies that have shown increased detection in vitreous samples and whole retinal tissues. Although conflicting reports have been published over the years, it is clear that alteration in growth factor expression is an important event that may in part be responsible for the development and progression of diabetic retinopathy to the proliferative stage. We will discuss the pathophysiological role of some specific growth factors that are believed to be involved in PDR.

### INSULIN-LIKE GROWTH FACTOR-I (IGF-I)

The IGFs, IGF-I and IGF-II, share considerable sequence homology with insulin (D'Amore, 1994). These growth factors are produced by a number of cell types, although liver seems to be the major contributor. These growth factors act by binding to cell surface receptors, IGF-IR and IGF-IIR (LeRoith and Roberts, 1993). IGF-IR binds IGF-I with higher affinity than IGF-II and insulin. IGF-IIR, on the other hand, preferentially binds IGF-II. Several circulating proteins, insulin-like growth factor binding proteins (IGFBPs), are responsible for the bioavailability and half-life of the IGFs (LeRoith and Roberts, 1993). The first indication as to the role of IGF-I in diabetic retinopathy came from a study in which hypophysectomy lead to a reduction in the severity of the diabetic eye condition (Poulsen, 1953). A possible role of growth hormone was promptly proposed following this observation as growth hormone mediates its effects via IGF-I. Consequently, the role of IGF-I in diabetic complications, including diabetic retinopathy has been extensively studied. There are contradictory reports as to the correlation between IGF-I and the clinical stage of diabetic retinopathy (Agardh et al., 1992; Boulton et al., 1997; Danis and Bingaman, 1997; Dills et al., 1991; Lee et al., 1994; Lowe et al., 1995; Meyer-Schwickerath et al., 1993; Pfeiffer et al., 1997; Wang et al., 1995). It should be noted that autocrine and paracrine tissue production of IGF-I might contribute to the inconsistency in correlation studies. In addition, when IGF-I levels are corrected for age, a significant increase in IGF-I levels in diabetic subjects is evident (Boulton et al., 1997). IGF-I is also increased in vitreous samples from patients with PDR (Merimee et al., 1983). In support of a possible connection between IGF-I and diabetic retinopathy, is the observation that administration of recombinant IGF-I in to the vitreous cavity of animals results in diabetic retinopathy–like pathologic changes (Thrailkill et al., 1999).

Functionally, IGF-I has been demonstrated to be angiogenic (King et al., 1985; Nakao-Hayashi et al., 1992; Nicosai et al., 1994). IGF-I has been shown to cause retinal capillary

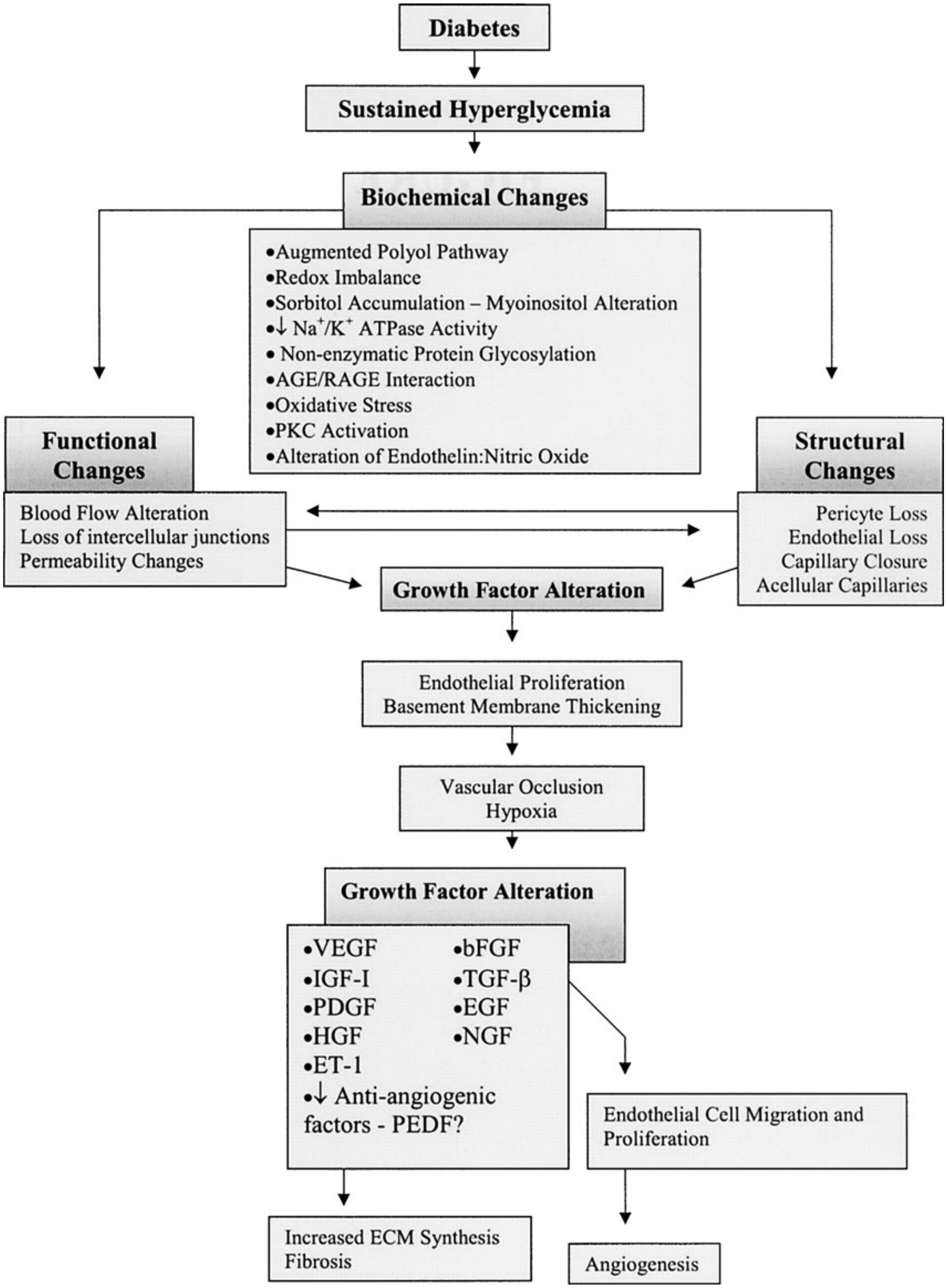

**FIGURE 1**

Schematic illustration of the pathogenesis of proliferative diabetic retinopathy. Chronic hyperglycemia leads to various biochemical abnormalities that ultimately lead to retinal ischemia and susceptibility to unregulated angiogenesis. Many growth factors are up-regulated along with their respective receptors. An imbalance between proangiogenic and antiangiogenic factors leads to new blood vessel formation in the retina. Due to the fragile nature of the newly formed blood vessels and inadequate cell-cell junctions, the vessels are prone to leakage and hemorrhage. The course of diabetic retinopathy ultimately leads to retinal and vitreous hemorrhage and retinal detachment.

endothelial cells to proliferate and migrate (Grant et al., 1987). Furthermore, implantation of IGF-I pellet in corneas of rabbits causes neovascularization (Grant et al., 1993). However, it is to be noted that the dose used in this study was significantly higher than that found in retinal and vitreous samples from patients with PDR. Interestingly, IGF-I is capable of increasing expression of VEGF in vivo and in vitro (Punglia et al., 1997). IGF-I can also increase transforming growth factor-$\beta$ bioavailability via increased PA activity (Grant et al., 1990). This suggests that IGF-I might be important in directing angiogenesis by itself and also by regulating various other growth factors.

### PLATELET-DERIVED GROWTH FACTOR (PDGF)

PDGFs are produced by a number of cells including platelets, endothelial cells, vascular smooth muscle cells, fibroblasts, and macrophages (Heldin and Westermark, 1999; Raines, 1993). There are three forms of this growth factor, which are produced by homo- and heterodimerization of two polypeptides, PDGF A and PDGF B. Two receptors for PDGF have been cloned (Heldin and Westermark, 1999; Raines, 1993). PDGF AA polypeptide binds to $\alpha$ receptors whereas PDGF BB polypeptide and PDGF heterodimer (PDGF AB) binds to both $\alpha$ and $\beta$ receptors. Similar to other growth factor receptors, PDGF receptors also posseses intrinsic tyrosine kinase activity. Vascular endothelial cells express both PDGF A and B polypeptides along with $\beta$ receptors (Bar et al., 1989; Dicorleto and Brown-Pope, 1983; Raines et al., 1990; Smits et al., 1989). Generally, $\beta$ receptors are involved in cell growth, which is the case with endothelial cells. Activation of $\beta$ receptors on endothelial cells leads to transduction of strong mitogenic signals (Battegay et al., 1994; Kuwabara et al., 1995; Raines et al., 1990).

Both in vivo and in vitro studies indicate that PDGF possesses an angiogenic property (Marx et al., 1994; Risau et al., 1992; Sato et al., 1993). PDGF levels have been shown to be upregulated in vitreous samples from patients with PDR (Cassidy et al., 1998; Endo et al., 2000; Freyberger et al., 2000). In addition, PDGF has been shown to augment VEGF expression in response to hypoxia (Stavri et al., 1995). Whether PDGF augments or aggravates the effects of angiogenic factors in the context of PDR, other than VEGF, remains to be determined. However, in a recent study, it was demonstrated that PDGF BB causes increased expression of endothelin-1 in bovine retinal pigment epithelial cells and endothelial cells, which was inhibited by a general PKC inhibitor (Yokota et al., 2003). PDGF is also believed to be important in the early pathogenetic changes in the retina. It has been demonstrated that PDGF ablation in endothelial cells produces morphological changes, such as microaneurysms and acellular capillaries (Enge et al., 2002), which are reminiscent of early vascular changes in diabetic retinopathy.

### BASIC FIBROBLAST GROWTH FACTOR (bFGF)

FGF is a heparin-binding peptide (Esch et al., 1985; Gospodarowicz et al., 1986; Sporn and Roberts, 1988). FGF is produced by a variety of cells, including fibroblasts, macrophages, and endothelial cells (Enge et al., 2002; Esch et al., 1985; Gospodarwicz et al., 1986; Schweigerer et al., 1987, 1988; Sporn and Roberts, 1988; Sternfeld et al., 1989). Two isoforms of FGFs have been cloned, acidic FGF (aFGF) and basic FGF (bFGF). These two isoforms share 53% sequence homology and possess mitogenic properties toward fibroblasts and endothelial cells (De Juan et al., 1990; Schweigerer et al., 1987). bFGF is found in nearly all tissues, whereas, aFGF is mainly present in neural tissues. Generally, bFGF is a protein localized to the ECM, bound to heparin, which protects it from degradation (Bashkin et al., 1989; Prestrelski et al., 1992; Saksela et al., 1988; Sommer and Rifkin, 1989). A lack of secretory signal on bFGF suggests that it might be introduced to the ECM by tissue damage or an exocytotic process (Gajdusek and Carbon, 1989; Muthukrishnan et al., 1991). Expression of FGF and its receptor in the retina prompted researchers to study the role of FGF in PDR in great detail (Baird et al., 1985; Hanneken et al., 1991; Khaliq et al., 1995).

In vivo angiogenesis assays have shown that bFGF is a very potent angiogenic factor (Glaser et al., 1980a, 1980b; Wong et al., 2001). It is also increased in vitreous samples from patients with PDR (Sivalingam et al., 1990). Exogenous administration of bFGF has been shown to induce endothelial proliferation and VEGF expression (Stavri et al., 1995). Both of these effects suggest important roles in retinal neovascularization. However, there still remains quite a controversy as to the exact in vivo role played by these factors. In vivo and in vitro studies have demonstrated increased bFGF expression in various systems. However, bFGF immunostaining has only been localized to mature nonproliferating blood vessel cells (Hanneken et al., 1991). In addition, hyperglycemia has been shown to cause glycosylation of bFGF (Giardino et al., 1994), which reduces its mitogenic activity. It is plausible that bFGF is involved in increased expression of other secreted angiogenic factors, such as VEGF, which promote proliferation of endothelial cells.

### TRANSFORMING GROWTH FACTOR-$\beta$ (TGF-$\beta$)

TGF-$\beta$ is a very important cytokine in fibrotic diseases of the liver, kidney, and lungs. TGF-$\beta$ is a member of a large family of regulatory proteins that include activin, inhibin, and bone morphogenic protein (Barnard et al., 1990; Ruscetti et al., 1998). Mammals express three TGF-$\beta$ isoforms ($\beta_1$, $\beta_2$, and $\beta_3$), which are encoded by separate genes. TGF-$\beta$ shows widespread tissue distribution and biological actions. In diabetic retinopathy, the role of TGF-$\beta$ seems to be complex. TGF-$\beta$ has been shown to be up-regulated by high glucose levels in human retinal endothelial cells (Pascal et al., 1999). In addition, TGF-$\beta_2$ levels were found to be increased in vitreous samples from patients with PDR (Hirase et al., 1998). Although, TGF-$\beta$ exihibits antiproliferative activity in vitro, it is implicated in a positive regulation of angiogenesis in vivo (Battegay, 1995; Beck and D'Amore, 1997; Bussolino et al., 1997; Folkman and Klagsburn, 1987; Merwin et al., 1991; Muller et al., 1987; Roberts et al., 1986; Yang and Moses, 1990). This discrepancy is the least understood aspect of TGF-$\beta$ action. Several theories have been proposed to reconcile these contradictory reports. The most attractive being the chemoattractant theory. According to this postulate, TGF-$\beta$ acts as a chemoattractant for various cell types, including fibroblasts and monocytes, both of which are capable of producing angiogenic factors such as VEGF, PDGF, and tumor necrosis factor (TNF)-$\alpha$ (Phillips et al., 1993; Sakamoto et al., 2000). Furthermore, some in vitro evidence exists that implicates expression of TNF-$\alpha$ in TGF-$\beta$–mediated angiogenesis (Iruela-Arispe and Sage, 1993; Vinals and Pouyssegur, 2001). This theory suggests an indirect role of TGF-$\beta$ in angiogenesis mediated either by bystander cells or by the induction of angiogenic factors. Further support for its involvement in PDR is the observation that TGF-$\beta$ modulates VEGF expression in endothelial cells (Pertovaara et al., 1994). Endothelial cell permeability is another aspect that is in part regulated by TGF-$\beta$ (Behzadian et al., 2001). It has been demonstrated that TGF-$\beta$ regulates retinal endothelial permeability by modulating proteases which degrade ECM proteins.

Interestingly, various other growth factors that are altered in chronic diabetic complications (VEGF, IGF-I, bFGF) increase the activity of PAs, which convert plasminogen to plasmin. Plasmin is involved in producing a conformational change in the latent TGF-$\beta$–binding protein, leading to release of TGF-$\beta$ in bioactive form. Increased TGF-$\beta$, in turn, can modulate ECM protein synthesis and degradation, a balance that is altered in diabetic retinopathy.

### VASCULAR ENDOTHELIAL GROWTH FACTOR (VEGF)

VEGFs are a family of growth factors with five isoforms that are produced from a single gene by alternative splicing (Robinson and Stringer, 2001). These growth factors are also produced by a variety of cells, including macrophages, vascular smooth muscle cells, vascular endothelial cells, and retinal pigment epithelial cells. These growth factors act on VEGF receptors (flt-1 and flk-1), which are mainly localized on vascular endothelial cells and probably to some extent on vascular smooth muscle cells (De Vries et al., 1992; Hewett and Murray, 1996; Terman et al., 1992). VEGF has been shown to be mitogenic for endothelial cells and its expression is increased in various animal models prior to neovascularization (Clermont et al., 1997; Favard et al., 1996; Gerhardinger et al., 1998; Grunwald, 1998; Lu and Adamis, 2002; Miller et al., 1997; Noma et al., 2002; Ohno-Matsui et al., 2002; Pe'er et al., 1996; Tanaka et al., 1997). In addition, it is up-regulated in hypoxic conditions and is also modulated by various other growth factors as mentioned earlier (Donahue et al., 1996; Marsh et al., 2000; Oh et al., 1999). It has been shown to be up-regulated in vitreous samples of patients with active PDR (Aiello et al., 1994; Endo et al., 2001; Funatsu et al., 2001; Mitamura et al., 2002; Ogata et al., 2002; Shinoda et al., 1999; Simo et al., 2002). This suggests that VEGF might be the perfect candidate to carry out signals for retinal neovascularization. A PKC-dependent pathway has been thought to mediate VEGF activation (Zhou et al., 2002). On the other hand, the mechanism by which VEGF carries out its action also seems to involve PKC activation. VEGF binding to the receptor results in increased phosphatidylinositol 3-kinase ($PI_3$ kinase) activity and phospholipase-C$\gamma$ (PLC$\gamma$) activation (Aiello et al., 1997; Gliki et al., 2002; Mathews et al., 1997; Seymour et al., 1996; Teicher et al., 2002; Wellner et al., 1999; Zhou et al., 2002). The subsequent increase in diacylglycerol (DAG) results in activation of PKC isoforms $\alpha$ and $\beta$. It has been shown that inhibition of PKC prevents VEGF-mediated increased permeability and proliferation of endothelial cells (Aiello et al., 1997; Gliki et al., 2002; Teicher et al., 2002; Wellner et al., 1999). Furthermore, downstream effector molecules of VEGF receptor–mediated signaling also include guanine 5′-triphosphate (GTP)ase-activating protein, in addition to PKC and $PI_3$ kinase (Qi and Claesson-Welsh, 2001; Suzuma et al., 2000; Takahashi and Shibuya, 1997).

In animal models, chimeric VEGF receptor proteins, which sequester VEGF and prevent it from binding to the receptors on cell surfaces, can suppress neovascularization in almost all animals studied (Aiello et al., 1995; Adamis et al., 1996; Ozaki et al., 2000). Similar results have been obtained in experiments utilizing antisense oligonucleotides against VEGF, which lead to in decreased VEGF production (Robinson et al., 1996). The use of antisense oligonucleotides resulted in suppression of neovascularization in almost 75% of animals studied. These findings certainly provide evidence of an integral role of VEGF in retinal neovascularization.

### ENDOTHELINS (ETs)

Endothelins are potent vasoactive peptides that regulate vascular tone by their action on vascular endothelial and smooth muscle cells (Levin, 1995; Rubanyi and Polokof, 1994). The ET family is comprised of three isoforms, ET-1, ET-2, and ET-3 (Inoue et al., 1989; Levin, 1995; Rubanyi and Polokof, 1994). These peptides are produced by a number of tissues. The major source of ET-1, the most potent vasoconstrictor, is vascular endothelium (Yanagisawa et al., 1988). Regulation of ETs is achieved in a transcription-dependent manner (Levin, 1995; Rubanyi and Polokof, 1994). These vasoactive peptides interact with specific cell surface receptors, $ET_A$, $ET_B$, and $ET_C$ (Sakurai et al., 1992; Sumner et al., 1992). Only $ET_A$ and $ET_B$ receptor types are present in mammals. These receptors are coupled to PLC through G proteins. $ET_A$ receptors are localized primarily on vascular smooth muscle cells and are involved in vasoconstriction (Sakurai et al., 1992; Sumner et al., 1992). Activation of $ET_A$ receptors results in calcium influx via PLC-mediated DAG and inositol trisphosphate production. $ET_B$ receptors are involved in generation of nitric oxide by endothelial cells and thus regulate vasodilation.

In addition to a number of inducing factors, tissue hypoxia and ischemia cause up-regulation of ETs (Blauw et al., 1995; Karmazyn, 1996). Alteration of ETs has been demonstrated in both type I and type II diabetes (Bertello et al., 1994; De Mattia et al., 1998; Donatelli et al., 1994; Haak et al., 1992; Kamoi et al., 1994; Kawamura et al., 1992; Laurenti et al., 1997; Letizia et al., 1997; Morise et al., 1995; Takahashi et al., 1990). Although, a number of studies can be cited that provide contradictory reports of plasma ET levels in diabetic patients, it should be noted that these peptides act in both an autocrine and paracrine fashion. Therefore plasma levels may not provide an adequate assessment of their biological activity in regards to the retina (Levin, 1995; Rubanyi and Polokof, 1994). In a recent study, vitreous ET-1 levels were found to be significantly elevated in patients with PDR as compared to patients with nondiabetes associated ocular conditions (Oku et al., 2001).

ETs exhibit mitogenic property on vascular endothelial cells and may be involved in diabetes-induced retinal neovascularization (Bek and McMillen, 2000; Brennan and Zaki, 2000; Chollet et al., 1993; Morbidelli et al., 1995; Salani et al., 2000). The mitogenic property was demonstrated in the early 1990s by DNA synthesis assays. Administration of ET-1 was shown to induce DNA synthesis in brain capillary endothelial cells (Vigne et al., 1990). In addition, it has been demonstrated that selective $ET_B$ receptor antagonist can prevent endothelial cell proliferation and migration, two fundamental steps in the process of angiogenesis. In further support of the role of ETs in the development and progression of PDR is the study from our laboratory that has demonstrated that ETs are involved in hyperglycemia-induced increased permeability (Chen et al., 2000). The downstream effector molecule regulating ET-mediated mitogenic effects and increased permeability seems to be PKC activation (Chen et al., 2000; Stanimirovic et al., 1994). This suggests a possible role of these vasoactive factors in mediating hyperglycemia-induced retinal structural and functional changes associated with PDR.

### PIGMENT EPITHELIUM-DERIVED FACTOR (PEDF)

Recent advances in diabetic retinopathy research have reframed our thinking in regards to the role of growth factors promoting angiogenesis. Initially, retinal neovascularization was thought to be the result of augmented growth factor expression, which promotes new blood vessel growth. It is now being accepted that pathologic angiogenesis in the eye is the result of not only increased vessel growth-stimulating factors but also decreased antiangiogenic factors. PEDF is such an antiangiogenic factor, which is believed to carry out its activity by inhibiting proliferation of endothelial cells (Dawson et al., 1999; Hutchings et al., 2002). PEDF has been shown to be present in the vitreous humor with high antiangiogenic activity, keeping this ocular portion avascular (Alberdi et al., 1999; Singh et al., 1998; Wu et al., 1995). Removal of PEDF from vitreous results in invasion of numerous blood vessels. In addition, induction of corneal wounds does not normally induce angiogenesis but does develop neovascular structure when facilitated with antibodies against PEDF (Dawson et al., 1999). The mechanism by which PEDF carries out its antiangiogenic activity is still not clear. However, a receptor has been isolated from retinoblastoma cells and also retinal neural cells (Alberdi et al., 1999; Becerra, 1997). Binding of PEDF to endothelial cells with high affinity also suggests the presence of a receptor, possibly similar to the one isolated from neural cells, on endothelial cells.

The role played by PEDF has not been fully elucidated in the context of retinal neovascularization. However, it has been recently shown that there is a negative correlation between PEDF levels and the degree of ischemia-induced neovasularization in rats (Gao et al., 2001). Low levels of PEDF have also been associated with angiogenic ocular diseases in humans (Ogata et al., 2001; Spranger et al., 2001). Furthermore, the antiangiogenic activity that is believed to be suppressed in retinal neovascularization can be recovered by administration of recombinant PEDF as determined by prevention of retinopathy (Duh et al., 2002; Mori et al., 2001; Stellmach et al., 2001). These observations suggest that an imbalance in angiogenic and antiangiogenic factors, produced by suppressed PEDF and augmented proangiogenic factors such as VEGF, promote new blood vessel formation in the retina in PDR.

### OTHER FACTORS

Apart from the growth factors that we have described so far, a number of potent factors, which by their angiogenic or other activities, are involved in diabetic retinopathy. We will briefly describe our current knowledge in regards to the involvement of such factors in PDR.

### Nerve Growth Factor (NGF)

NGF belongs to the neurotrophin family of polypeptides that play important roles in survival of neurons in both central and peripheral nervous system (Greene and Shooter, 1980). Recent studies have indicated that NGF and NGF-mediated signaling might be important in other biological functions, including wound healing and inflammation (Lawman et al., 1985; Matsuda et al., 1998). NGF has also been demonstrated to promote proliferation of microvascular endothelial cells (Raychaudhuri et al., 2001). This suggests that NGF alteration might play an important role in the progression of diabetic retinopathy. Although no difference has been observed in NGF and NGF receptor immunoreactivity between normal and diabetic retina in spontaneously diabetic BB rats (Chakrabarti et al., 1990), the idea cannot be excluded that some alteration of NGF takes place in the retina of diabetic subjects. A more recent study has demonstrated increased serum levels of NGF in patients with type I diabetes compared to age-matched control subjects and type II patients (Azar et al., 1999). In addition, several other growth factors that are altered in long-standing diabetes, such as b-FGF and epidermal growth factor (EGF), can induce the expression of NGF by fibroblasts and possibly other cells (Hattori et al., 1993).

### Hepatocyte Growth Factor (HGF)

HGF was initially isolated from serum of hepatectomized rats (Nakamura et al., 1984). This growth factor is produced by many mesenchymal cells including fibroblasts and endothelial cells. HGF has been shown to promote endothelial growth and migration and the formation of blood vessels in vivo (Bussolino et al., 1992; Cai et al., 2000; Grant et al., 1993). Only recently, have alterations in the vitreous levels of HGF been demonstrated in patients with diabetes-induced retinopathy (Nishimura et al., 1999, 2000). The levels of HGF in vitreous samples parallel the severity of PDR as determined by vitreous hemorrhage, fibrovascular proliferation, and tractional retinal detachment. In addition, the levels of HGF found in this study were in the range that has previously been shown to induce growth and proliferation of cultured endothelial cells (Nakamura et al., 1996). Although HGF is not as aggresively studied as other factors, its contributions to the development of PDR should not be ruled out.

### Epidermal Growth Factor (EGF)

EGF is a highly mitogenic polypeptide produced by a variety of cell types (Carpenter, 1985; Lui and Grandis, 2002). A number of studies have concluded that EGF is mitogenic for corneal endothelial cells and is involved in corneal neovascularization (Nezu et al., 1992; Woost et al., 1992). However, the involvement of EGF and its possible role in regulating retinal angiogenesis is less characterized compared to this function by other growth factors. One recent study has shown increased EGF and EGF receptor immunoreactivity in retinal samples from subjects with PDR (Patel et al., 1994). Although EGF possesses limited angiogenic activity as compared to FGF, it is highly mitogenic for human retinal pigment epithelial cells and may act synergistically with FGF (Leschey et al., 1990). This suggests that EGF signaling might be involved in preretinal membrane formation. This finding, along with localization of EGF in preretinal membranes of patients with PDR (Fredj-Reygrobellet et al., 1991; Patel et al., 1994), points to the involvement of EGF in the progression of diabetic retinopathy.

### Tumor Necrosis Factor-$\alpha$ (TNF-$\alpha$)

TNF-$\alpha$ is a proinflammatory cytokine primarily produced by phagocytes (Pober and Cotran, 1990). The principle target of TNF-$\alpha$ is vascular endothelium (Madge and Pober, 2001; Pober, 1998). The mechanistic basis of TNF-$\alpha$–mediated endothelial activation is not completely understood. However, accumulating evidence suggests that TNF-$\alpha$ may mediate alteration of vasoregulation and leukocyte adhesion, leading to endothelial dysfunction. In addition, it is well documented that TNF-$\alpha$ increases endothelial permeability (Friedl et al., 2002; Mark and Miller, 1999), an activity important in cell invasion and migration during angiogenesis. Vitreous and serum from diabetic subjects with PDR do show an elevated level of TNF-$\alpha$ (Doganay et al., 2002; Spranger et al., 1995; Yuuki et al., 2001). In addition, in vitro studies have demonstrated a potential role of TNF-$\alpha$ in TGF-$\beta$–induced angiogenesis (Iruela-Arispe and Sage, 1993; Vinals and Pouyssegur, 2001). The stage of diabetic retinopathy at which TNF-$\alpha$ might play a role is still obscure. However, mere elevation of this proinflammatory cytokine in PDR suggests an association that might be worth pursuing.

### CONCLUDING REMARKS

PDR is a very complex condition that results from altered expression of various growth factors and vasoactive factors. In the retina, hyperglycemia causes several metabolic defects, including oxidative stress, nonenzymatic glycation, and formation of advanced glycation end (AGE) products, sorbitol/*myo*-inositol–mediated changes, and redox potential alteration. These events set the background for pericyte loss, basement membrane thickening, microaneurysms, acellular capillaries, nonperfusion, ischemia and, therefore, susceptibility towards mitogenic stimuli. The increase in angiogenic factors or inducers of these factors results in a propagating complex that interplays between different pathways, resulting in nonregulated angiogenesis. In addition, hyperinsulinemia due to insulin resistance may also influence growth factor alteration. It is to be noted that an obligatory role of a particular growth factor in the development and progression of diabetic retinopathy is controversial. Our current knowledge suggests that a very heterogenous pattern of growth factor expression, with cross-talks between different pathways resulting in ocular neovascularization.

### REFERENCES

Adamis, A. P., Shima, D. T., Tolentino, M. J., Gragoudas, E. S., Ferrara, N., Folkman, J., D'Amore, P. A., and Miller, J. W. (1996) Inhibition of vascular endothelial growth factor prevents retinal ischemia-associated iris neovascularization in a nonhuman primate. *Arch. Ophthalmol.,* **114,** 66–71.

Agardh, C. D., Agardh, E., Eckert, B., and Sjoberg, U. (1992) Growth hormone levels in the basal state and after thyrotropin-releasing hormone stimulation in young type 1 (insulin-dependent) diabetic patients with severe retinopathy. *Diabetes. Res.,* **19,** 81–85.

Aiello, L. P., Avery, R. L., Arrigg, P. G., Keyt, B. A., Jampel, H. D., Shah, S. T, Pasquale, L. R., Thieme, H., Iwamoto, M. A., Park, J. E., Nguyen, H. V., Aiello, L. M., Ferrara, N., and King, G. L. (1994) Vascular endothelial growth factor in ocular fluid of patients with diabetic retinopathy and other retinal disorders. *N. Engl. J. Med.,* **331,** 1480–1487.

Aiello, L. P., Bursell, S. E., Clermont, A., Duh, E., Ishii, H., Takagi, C., Mori, F., Ciulla, T. A., Ways, K., Jirousek, M., Smith, L. E., and King, G. L. (1997) Vascular endothelial growth factor-induced retinal permeability is mediated by protein kinase C in vivo and suppressed by an orally effective beta-isoform-selective inhibitor. *Diabetes,* **46,** 1473–1480.

Aiello, L. P., Pierce, E. A., Foley, E. D., Takagi, H., Chen, H., Riddle, L., Ferrara, N., King, G. L., and Smith, L. E. (1995) Suppression of retinal neovascularization in vivo by inhibition of vascular endothelial growth factor (VEGF) using soluble VEGF-receptor chimeric proteins. *Proc. Natl. Acad. Sci. U. S. A.,* **92,** 10457–10461.

Alberdi, E., Aymerich, M. S., and Becerra, S. P. (1999) Binding of pigment-epithelium derived factor (PEDF) to retinoblastoma cells and cerebellar granule neurons. *J. Biol. Chem.,* **274,** 31605–31612.

Alder, V. A., Su, E. N., Yu, D. Y., Cringle, S. J., and Yu, P. K. (1997) Diabetic retinopathy: Early functional changes. *Clin. Exp. Pharmacol. Physiol.,* **24,** 785–788.

Azar, S. T., Major, S. C., and Safieh-Garabedian, B. (1999) Altered plasma levels of nerve growth factor and transforming growth factor-beta2 in type-1 diabetes mellitus. *Brain. Behav. Immunol.,* **13,** 361–366.

Baird, A., Esch, F., Gospodarowicz, D., and Guillemin, R. (1985) Retina- and eye-derived endothelial cell growth factors: Partial molecular characterization and identity with acidic and basic fibroblast growth factors. *Biochemistry,* **24,** 7855–7860.

Bar, R. S., Boes, M., Booth, B. A., Dake, B. L., Henley, S., and Hart, M. N. (1989) The effects of platelet-derived growth factor in cultured microvessel endothelial cells. *Endocrinology,* **124,** 1841–1848.

Barnard, J. A., Lyons, R. M., and Moses, H. L. (1990) The cell biology of transforming growth factor beta. *Biochim. Biophys. Acta,* **1032,** 79–87.

Bashkin, P., Doctrow, S., Klagsbrun, M., Svahn, C. M., Folkman, J., and Vlodavsky, I. (1989) Basic fibroblast growth factor binds to subendothelial extracellular matrix and is released by heparitinase and heparin-like molecules. *Biochemistry,* **28,** 1737–1743.

Battegay, E. J. (1995) Angiogenesis: Mechanistic insights, neovascular diseases, and therapeutic prospects. *J. Mol. Med.,* **73,** 333–346.

Battegay, E. J., Rupp, J., Iruela-Arispe, L., Sage, E. H., and Pech, M. (1994) PDGF-BB modulates endothelial proliferation and angiogenesis in vitro via PDGF beta-receptors. *J. Cell Biol.,* **125,** 917–928.

Becerra, S. P. (1997) Structure-function studies on PEDF. A noninhibitory serpin with neurotrophic activity. *Adv. Exp. Med. Biol.,* **425,** 223–237.

Beck, L., Jr., and D'Amore, P. A. (1997) Vascular development: Cellular and molecular regulation. *FASEB J.,* **11,** 365–373.

Behzadian, M. A., Wang, X. L., Windsor, L. J., Ghaly, N., and Caldwell, R. B. (2001) TGF-$\beta$ increases retinal endothelial cell permeability by increasing MMP-9: Possible role of glial cells in endothelial barrier function. *Invest. Ophthalmol. Vis. Sci.,* **42,** 853–859.

Bek, E. L., and McMillen, M. A. (2000) Endothelins are angiogenic. *J. Cardiovasc. Pharmacol.,* **36,** S135–S139.

Bertello, P., Veglio, F., Pinna, G., Gurioli, L., Molino, P., Alban, S., and Chiandussi, L. (1994) Plasma endothelin in NIDDM patients with and without complications. *Diabetes Care,* **17,** 574–577.

Bierhaus, A., Hofmann, M. A., Ziegler, R., and Nawroth, P. P. (1998) AGEs and their interaction with AGE-receptors in vascular disease and diabetes mellitus. I. The AGE concept. *Cardiovasc. Res.,* **37,** 586–600.

Blauw, G. J., Westendorp, R. G., Srivistava, N., Burggraaf, K., Frolich, M., Simons, R., Cohen, A. F., and Meinders, A. E. (1995) Hypoxia induced arterial endothelin does not influence peripheral vascular tone. *J. Cardiovasc. Pharmacol.,* **26,** S242–S243.

Boulton, M., Gregor, Z., McLeod, D., Charteris, D., Jarvis-Evans, J., Moriarty, P., Khaliq, A., Foreman, D., Allamby, D., and Bardsley, B. (1997) Intravitreal growth factors in proliferative diabetic retinopathy: Correlation with neovascular activity and glycaemic management. *Br. J. Ophthalmol.,* **81,** 228–233.

Brennan, P. A., and Zaki. G. A. (2000) Angiogenesis in cancer: The role of endothelin-1. *Ann. R. Coll. Surg. Engl.,* **82,** 363–364.

Brownlee, M. (2001) Biochemistry and molecular cell biology of diabetic complications. *Nature,* **414,** 813–820.

Brownlee, M., Cerami, A., and Vlassara, H. (1988) Advanced glycosylation end products in tissue and the biochemical basis of diabetic complications. *N. Engl. J. Med.,* **318,** 1315–1321.

Bussolino, F., Di Renzo, M. F., Ziche, M., Bocchietto, E., Olivero, M., Naldini, L., Gaudino, G., Tamagnone, L., Coffer, A., and Comoglio, P. M. (1992) Hepatocyte growth factor is a potent angiogenic factor which stimulates endothelial cell motility and growth. *J. Cell Biol.,* **119,** 629–641.

Bussolino, F., Mantovani, A., and Persico, G. (1997) Molecular mechanisms of blood vessel formation. *Trends. Biochem. Sci.,* **22,** 251–256.

Cai, W., Rook, S. L., Jiang, Z. Y., Takahara, N., and Aiello, L. P. (2000) Mechanisms of hepatocyte growth factor-induced retinal endothelial cellmigration and growth. *Invest. Ophthalmol. Vis. Sci.,* **41,** 1885–1893.

Carpenter, G. (1985) Epidermal growth factor: Biology and receptor metabolism. *J. Cell Sci.,* **3,** 1–9.

Casey, R., and Li, W. W. (1997) Factors controlling ocular angiogenesis. *Am. J. Ophthalmol.,* **124,** 521–524.

Cassidy, L., Barry, P., Shaw, C., Duffy, J., and Kennedy, S. (1998) Platelet derived growth factor and fibroblast growth factor basic levels in the vitreous of patients with vitreoretinal disorders. *Br. J. Ophthalmol.,* **82,** 181–185.

Chakir, M., and Plante, G. E. (1996) Endothelial dysfunction in diabetes mellitus. *Prostaglandins Leukot. Essent. Fatty Acids,* **54,** 45–51.

Chakrabarti, S., Sima, A. A., Lee, J., Brachet, P., and Dicou, E. (1990) Nerve growth factor (NGF), proNGF and NGF receptor-like immunoreactivity in BB rat retina. *Brain Res.,* **523,** 11–15.

Chen, S., Apostolova, M. D., Cherian, M. G., and Chakrabarti, S. (2000) Interaction of endothelin-1 with vasoactive factors in mediating glucose-induced increased permeability in endothelial cells. *Lab. Invest.,* **80,** 1311–1321.

Chollet, P., Malecaze, F., Gouzi, L., Arne, J. L., and Plouet, J. (1993) Endothelin 1 is a growth factor for corneal endothelium. *Exp. Eye Res.,* **57,** 595–600.

Clermont, A. C., Aiello, L. P., Mori, F., Aiello, L. M., and Bursell, S. E. (1997) Vascular endothelial growth factor and severity of nonproliferative diabetic retinopathy mediate retinal hemodynamics in vivo: A potential role for vascular endothelial growth factor in the progression of nonproliferative diabetic retinopathy. *Am. J. Ophthalmol.,* **124,** 433–446.

Costantino, L., Rastelli, G., Vianello, P., Cignarella, G., and Barlocco, D. (1999) Diabetes complications and their potential prevention: Aldose reductase inhibition and other approaches. *Med. Res. Rev.,* **19,** 3–23.

D'Amore, P. A. (1994) Mechanisms of retinal and choroidal neovascularization. *Invest. Ophthalmol. Vis. Sci.,* **35,** 3974–3979.

Danis, R. P., and Bingaman, D. P. (1997) Insulin-like growth factor-1 retinal microangiopathy in the pig eye. *Ophthalmology,* **104,** 661–669.

Davis, M. D. (1992) Diabetic retinopathy. A clinical overview. *Diabetes Care,* **15,** 1844–1874.

Dawson, D. W., Volpert, O. V., Gillis, P., Crawford, S. E., Xu, H., Benedict, W., and Bouck, N. P. (1999) Pigment epithelium-derived factor: A potent inhibitor of angiogenesis. *Science,* **285,** 245–248.

De Juan, E., Stefansson, E., and Ohira, A. (1990) Basic fibroblast growth factor stimulates 3H-thymidine uptake in retinal venular and capillary endothelial cells in vivo. *Invest. Ophthalmol. Vis. Sci.,* **31,** 1238–1244.

De Mattia, G., Cassone-Faldetta, M., Bellini, C., Bravi, M. C., Laurenti, O., Baldoncini, R., Santucci, A., and Ferri, C. (1998) Role of plasma and urinary endothelin-1 in early diabetic and hypertensive nephropathy. *Am. J. Hypertens.,* **11,** 983–988.

De Vries, C., Escobedo, J. A., Ueno, H., Houck, K., Ferrara, N., and Williams, L. T. (1992) The fms-like tyrosine kinase, a receptor for vascular endothelial growth factor. *Science,* **255,** 989–991.

De Vriese, A. S., Verbeuren, T. J., Van de Voorde, J., Lameire, N. H., and Vanhoutte, P. M. (2000) Endothelial dysfunction in diabetes. *Br. J. Pharmacol.,* **130,** 963–974.

Derubertis, F. R., and Craven, P. A. (1994) Activation of protein kinase C in glomerular cells in diabetes. Mechanisms and potential links to the pathogenesis of diabetic glomerulopathy. *Diabetes,* **43,** 1–8.

Diabetes Control and Complications Trial Research Group. (1993) The effect of intensive treatment of diabetes on the development and progression of long-term complications in insulin-dependent diabetes mellitus. *N. Engl. J. Med.,* **29,** 977–986.

Dicorleto, P. E., and Brown-Pope, D. F. (1983) Cultured endothelial cells produce a platelet-derived growth factor-like protein. *Proc. Natl. Acad. Sci. U. S. A.,* **80,** 1919–1923.

Dills, D. G., Moss, S. E., Klein, R., and Klein, B. E. (1991) Association of elevated IGF-I levels with increased retinopathy in late-onset diabetes. *Diabetes,* **40,** 1725–1730.

Doganay, S., Evereklioglu, C., Er, H., Turkoz, Y., Sevinc, A., Mehmet, N., and Savli, H. (2002) Comparison of serum NO, TNF-alpha, IL-1beta, sIL-2R, IL-6 and IL-8 levels with grades of retinopathy in patients with diabetes mellitus. *Eye,* **16,** 163–170.

Donahue, M. L., Phelps, D. L., Watkins, R. H., LoMonaco, M. B., and Horowitz, S. (1996) Retinal vascular endothelial growth factor (VEGF) mRNA expression is altered in relation to neovascularization in oxygen induced retinopathy. *Curr. Eye Res.,* **15,** 175–184.

Donatelli, M., Colletti, I., Bucalo, M. L., Russo, V., and Verga, S. (1994) Plasma endothelin levels in NIDDM patients with macroangiopathy. *Diabetes Res.,* **25,** 159–164.

Duh, E. J., Yang, H. S., Suzuma, I., Miyagi, M., Youngman, E., Mori, K., Katai, M., Yan, L., Suzuma, K., West, K., Davarya, S., Tong, P., Gehlbach, P., Pearlman, J., Crabb, J. W., Aiello, L. P., Campochiaro, P. A., and Zack, D. J. (2002) Pigment epithelium-derived factor suppresses ischemia-induced retinal neovascularization and VEGF-induced migration and growth. *Invest. Ophthalmol. Vis. Sci.,* **43,** 821–829.

Endo, H., Naito, T., Asahara, T., Kajima, M., and Shiota, H. (2000) Cytokines in the vitreous fluid of patients with proliferative diabetic retinopathy-vascular endothelial growth factor and platelet-derived growth factor are elevated in proliferative diabetic retinopathy. *Nippon Ganka Gakkai Zasshi,* **104,** 711–716.

Endo, M., Yanagisawa, K., Tsuchida, K., Okamoto, T., Matsushita, T., Higuchi, M., Matsuda, A., Takeuchi, M., Makita, Z., and Koike, T. (2001) Increased levels of vascular endothelial growth factor and advanced glycation end products in aqueous humor of patients with diabetic retinopathy. *Horm. Metab. Res.,* **33,** 317–322.

Enge, M., Bjarnegard, M., Gerhardt, H., Gustafsson, E., Kalen, M., Asker, N., Hammes, H. P., Shani, M., Fassler, R., and Betsholtz, C. (2002) Endothelium-specific platelet-derived growth factor-B ablation mimics diabetic retinopathy. *EMBO J.,* **21,** 4307–4316.

Engerman, R., Bloodworth, J. M., Jr., and Nelson, S. (1985) Relationship of microvascular disease in diabetes to metabolic control. *Diabetes,* **26,** 760–769.

Engerman, R. L., Kern, T. S., and Garment, M. B. (1993) Capillary basement membrane in retina, kidney, and muscle of diabetic dogs and galactosemic dogs and its response to 5 years aldose reductase inhibition. *J. Diabetes Complications,* **7,** 241–245.

Esch, F., Baird, A., Ling, N., Ueno, N., Hill, F., Denoroy, L., Klepper, R., Gospodarowicz, D., Bohlen, P., and Guillemin, R. (1985) Primary structure of bovine pituitary basic fibroblast growth factor (FGF) and comparison with the amino-terminal sequence of bovine brain acidic FGF. *Proc. Natl. Acad. Sci. U. S. A.,* **82,** 6507–6511.

Favard, C., Ortega, N., Bayard, F., and Plouet, J. (1996) Vascular endothelial growth factor and retinal neovascularisation: A new therapeutic approach for diabetic retinopathy. *Diabetes Metab.,* **22,** 268–273.

Folkman, J., and Klagsburn, M. (1987) Angiogenic factors. *Science,* **235,** 442–447.

Fong, D. S., Aiello, L., Gardner, T. W., King, G. L., Blankenship, G., Cavallerno, J. D., Ferris, F. L., 3rd, and Klein, R. (2003) Diabetic retinopathy. *Diabetes Care,* **26,** S99–S102.

Fredj-Reygrobellet, D., Baudouin, C., Negre, F., Caruelle, J. P., Gastaud, P., and Lapalus, P. (1991) Acidic FGF and other growth factors in preretinal membranes from patients with diabetic retinopathy and proliferative vitreoretinopathy. *Ophthalmic Res.,* **23,** 154–161.

Freyberger, H., Brocker, M., Yakut, H., Hammer, J., Effert, R., Schifferdecker, E., Schatz, H., and Derwahl, M. (2000) Increased levels of platelet-derived growth factor in vitreous fluid of patients with proliferative diabetic retinopathy. *Exp. Clin. Endocrinol. Diabetes,* **108,** 106–109.

Friedl, J., Puhlmann, M., Bartlett, D. L., Libutti, S. K., Turner, E. N., Gnant, M. F., and Alexander, H. R. (2002) Induction of permeability across endothelial cell monolayers by tumor necrosis factor (TNF) occurs via a tissue factor-dependent mechanism: Relationship between the procoagulant and permeability effects of TNF. *Blood,* **100,** 1334–1339.

Funatsu, H., Yamashita, H., Shimizu, E., Kojima, R., and Hori, S. (2001) Relationship between vascular endothelial growth factor and interleukin-6 in diabetic retinopathy. *Retina,* **21,** 469–477.

Gajdusek, C. M., and Carbon, S. (1989) Injury-induced release of basic fibroblast growth factor from bovine aortic endothelium. *J. Cell Physiol.,* **139,** 570–579.

Gao, G., Li, Y., Zhang, D., Gee, S., Crosson, C., and Ma, J. (2001) Unbalanced expression of VEGF and PEDF in ischemia-induced retinal neovascularization. *FEBS Lett.,* **489,** 270–276.

Gerhardinger, C., Brown, L. F., Roy, S., Mizutani, M., Zucker, C. L., and Lorenzi, M. (1998) Expression of vascular endothelial growth factor in the human retina and in nonproliferative diabetic retinopathy. *Am. J. Pathol.,* **152,** 1453–1462.

Giardino, I., Edelstein, D., and Brownlee, M. (1994) Nonenzymatic glycosylation in vitro and in bovine endothelial cells alters basic fibroblast growth factor activity. *J. Clin. Invest.,* **94,** 110–117.

Glaser, B. M., D'Amore, P. A., Michels, R. G., Brunson, S. K., Fenselau, A. H., Rice, T., and Patz, A. (1980a) The demonstration of angiogenic activity from ocular tissues. Preliminary report. *Ophthalmology,* **87,** 440–446.

Glaser, B. M., D'Amore, P. A., Michels, R. G., Patz, A., and Fenselau, A. (1980b). Demonstration of vasoproliferative activity from mammalian retina. *J. Cell Biol.,* **84,** 298–304.

Gliki, G., Wheeler-Jones, C., and Zachary, I. (2002) Vascular endothelial growth factor induces protein kinase C (PKC)-dependent Akt/PKB activation and phosphatidylinositol 3′-kinase-mediates PKC delta phosphorylation: Role of PKC in angiogenesis. *Cell Biol. Int.,* **26,** 751–759.

Gospodarowicz, D., Neufeld, G., and Schweigerer, L. (1986) Fibroblast growth factor. *Mol. Cell Endocrinol.,* **46,** 187–204.

Grant, D. S., Kleinman, H. K., Goldberg, I. D., Bhargava, M. M., Nickoloff, B. J., Kinsella, J. L., Polverini, P., and Rosen, E. M. (1993) Scatter factor induces blood vessel formation in vivo. *Cell Biol.,* **90,** 1937–1941.

Grant, M., Guay, C., and Marsh, R. (1990) Insulin-like growth factor-I stimulates proliferation, migration, and plasminogen activator release by human retinal pigment epithelial cells. *Curr. Eye Res.,* **9,** 323–335.

Grant, M., Jerdan, J., and Merimee, T. (1987) Insulin-like growth factor-I modulates endothelial cell chemotaxis. *J. Clin. Endocrinol. Metab.,* **65,** 370–371.

Grant, M. B., Mames, R. N., Fitzgerald, C., Ellis, E. A., Aboufriekha, M., and Guy, J. (1993) Insulin-like growth factor I acts as angiogenic agent in rabbit cornea and retina: comparative studies with basic fibroblast growth factor. *Diabetologia,* **36,** 282–291.

Greene, D. A., Lattimer, S. A., and Sima, A. A. (1987) Sorbitol, phosphoinositides, and sodium-potassium-ATPase in the pathogenesis of diabetic complications. *N. Engl. J. Med.,* **316,** 599–606.

Greene, L. A., and Shooter, E. M. (1980) The nerve growth factor: Biochemistry, synthesis, and mechanism of action. *Annu. Rev. Neurosci.,* **3,** 353–402.

Gross, J. L., Moscatelli, D., and Rifkin, D. B. (1983) Increased capillary endothelial cell protease activity in response to angiogenic stimuli. *Proc. Natl. Acad. Sci. U. S. A.,* **80,** 2623–2627.

Grunwald, J. E. (1998) Vascular endothelial growth factor and severity of nonproliferative diabetic retinopathy mediate retinal hemodynamics in vivo: A potential role for vascular endothelial growth factor in the progression of nonproliferative diabetic retinopathy. *Am. J. Ophthalmol.,* **125,** 731–732.

Haak, T., Jungmann, E., Felber, A., Hillmann, U., and Usadel, K. H. (1992) Increased plasma levels of endothelin in diabetic patients with hypertension. *Am. J. Hypertens.,* **5,** 161–166.

Hanneken, A., de Juan, E., Jr., Lutty, G. A., Fox, G. M., Schiffer, S., and Hjelmeland, L. M. (1991) Altered distribution of basic fibroblast growth factor in diabetic retinopathy. *Arch. Ophthalmol.,* **109,** 1005–1011.

Hattori, A., Tanaka, E., Murase, K., Ishida, N., Chatani, Y., Tsujimoto, M., Hayashi, K., and Kohno, M. (1993) Tumor necrosis factor stimulates the synthesis and secretion of biologically active nerve growth factor in non-neuronal cells. *J. Biol. Chem.,* **268,** 2577–2582.

Heldin, C. H., and Westermark, B. (1999) Mechanism of action and in vivo role of platelet-derived growth factor. *Physiol. Rev.,* **79,** 1283–1316.

Hewett, P. W., and Murray, J. C. (1996) Coexpression of flt-1, flt-4 and KDR in freshly isolated and cultured human endothelial cells. *Biochem. Biophys. Res. Commun.,* **221,** 697–702.

Hink, U., Li, H., Mollnau, H., Oelze, M., Matheis, E., Hartmann, M., Skatchkov, M., Thaiss, F., Stahl, R. A., Warnholtz, A., Meinertz, T., Griendling, K., Harrison, D. G., Forstermann, U., and Munzel, T. (2001) Mechanisms underlying endothelial dysfunction in diabetes mellitus. *Circ. Res.,* **88,** E14–E22.

Hirase, K., Ikeda, T., Sotozono, C., Nishida, K., Sawa, H., and Kinoshita, S. (1998) Transforming growth factor beta2 in vitreous in proliferative diabetic retinopathy. *Arch. Ophthalmol.,* **116,** 738–741.

Hudson, C. (1996) The clinical features and classification of diabetic retinopathy. *Ophthalmic Physiol. Opt.,* **16,** S43–S48.

Hutchings, H., Maitre-Boube, M., Tombran-Tink, J., and Plouet, J. (2002) Pigment epithelium-derived factor exerts opposite effects on endothelial cells of different phenotypes. *Biochem. Biophys. Res. Commun.,* **294,** 764–769.

Ido, Y., Kilo, C., and Williamson, J. R. (1997) Cytosolic $NADH/NAD^+$, free radicals and vascular dysfunction in early diabetes mellitus. *Diabetologia,* **40,** S115–S117.

Inoue, A., Yanagisawa, M., Kimura, S., Kasuya, Y., Miyauchi, T., Goto, K., and Masaki, T. (1989) The human endothelin family: Three structurally and pharmacologically distinct isopeptides predicted by three separate genes. *Proc. Natl. Acad. Sci. U. S. A.,* **86,** 2863–2867.

Iruela-Arispe, M. L., and Sage, E. H. (1993) Endothelial cells exhibit angiogenesis in vitro proliferate in response to TGF-beta1. *J. Cell Biochem.,* **52,** 414–430.

Ishii, H., Jirousek, M. R., Koya, D., Takagi, C., Xia, P., Clermont, A., Bursell, S. E., Kern, T. S., Ballas, L. M., Heath, W. F., Stramm, L. E., Feener, E. P., and King, G. L. (1996) Amelioration of vascular dysfunctions in diabetic rats by an oral PKC beta inhibitor. *Science,* **272,** 728–731.

Ishizuka, T., Hoffman, J., Cooper, D. R., Watson, J. E., Pushkin, D. B., and Farese, R. V. (1989) Glucose-induced synthesis of diacylglycerol de novo is associated with translocation (activation) of protein kinase C in rat adipocytes. *FEBS Lett.,* **249,** 234–238.

Johannes, F. J., Prestle, J., Eis, S., Oberhagemann, P., and Pfizenmaier, K. (1994) PKC is a novel, atypical member of the protein kinase C family. *J. Biol. Chem.,* **269,** 6140–6148.

Kamoi, K., Ishibashi, M., and Yamaji, T. (1994) Endothelin-1 and big endothelin-1 in NIDDM patients with and without microangiopathy. *Diabetes Res. Clin. Pract.,* **24,** 125–129.

Karmazyn, M. (1996) The role of endothelin in cardiac function in health and disease. In: *Myocardial Ischemia, Mechanisms, Reperfusion, Protection,* Edited by Karmazyn, M., pp. 209–230. Basel, Switzerland, Birkhauser.

Kawamura, M., Ohgawara, H., Naruse, M., Suzuki, N., Iwasaki, N., Naruse, K., Hori, S., Demura, H., and Omori, Y. (1992) Increased plasma endothelin in NIDDM patients with retinopathy. *Diabetes Care,* **15,** 1396–1397.

Kern, T. S., and Engerman, R. L. (2001) Pharmacological inhibition of diabetic retinopathy: Aminoguanidine and aspirin. *Diabetes,* **50,** 1636–1642.

Khaliq, A., Patel, B., Jarvis-Evans, J., Moriarty, P., McLeod, D., and Boulton, M. (1995) Oxygen modulates production of bFGF and TGF-beta by retinal cells in vitro. *Exp. Eye Res.,* **60,** 415–423.

King, G. L., and Brownlee, M. (1996) The cellular and molecular mechanisms of diabetic complications. *Endocrinol. Metab. Clin. North Am.,* **25,** 255–270.

King, G. L., Goodman, A. D., Buzney, S., Moses, A., and Kahn, C. R. (1985) Receptors and growth-promoting effects of insulin and insulinlike growth factors on cells from bovine retinal capillaries and aorta. *J. Clin. Invest.,* **75,** 1028–1036.

King, G. L., Shiba, T., Oliver, J., Inoguchi, T., and Bursell, S. E. (1994) Cellular and molecular abnormalities in the vascular endothelium of diabetes mellitus. *Annu. Rev. Med.,* **45,** 179–188.

Koya, D., and King, G. L. (1998) Protein kinase C activation and the development of diabetic complications. *Diabetes,* **47,** 859–866.

Kuwabara, K., Ogawa, S., Matsumoto, M., Koga, S., Clauss, M., Pinsky, D. J., Lyn, P., Leavy, J., Witte, L., Joseph-Silverstein, J., Furie, M. B., Torcia, G., Cozzolino, F., Kamada, T., and Stern, D. M. (1995) Hypoxia-mediated induction of acidic/basic fibroblast growth factor and platelet-derived growth factor in mononuclear phagocytes stimulates growth of hypoxic endothelial cells. *Proc. Natl. Acad. Sci. U. S. A.,* **9,** 4606–4610.

Laurenti, O., Vingolo, E. M., Desideri, G. B., Ferri, C., Bellini, C., Cassone-Faldetta, M., Santucci, A., and De Mattia, G. (1997) Increased levels of plasma endothelin-1 in non-insulin dependent diabetic patients with retinopathy but without other diabetes-related organ damage. *Exp. Clin. Endocrinol. Diabetes,* **105,** 40–42.

Lawman, M. J., Boyle, M. D., Gee, A. P., and Young, M. (1985) Nerve growth factor accelerates the early cellular events associated with wound healing. *Exp. Mol. Pathol.,* **43,** 274–281.

Lee, H. C., Lee, K. W., Chung, C. H., Chung, Y. S., Lee, E. J., Lim, S. K., Kim, K. R., Huh, K. B., Lee, S. C., and Kwon, O. W. (1994) IGF-I of serum and vitreous fluid in patients with diabetic proliferative retinopathy. *Diabetes Res. Clin. Pract.,* **24,** 85–88.

LeRoith, D., and Roberts, C., Jr. (1993) Insulin-like growth factors. *Ann. N. Y. Acad. Sci.,* **692,** 1–9.

Leschey, K. H., Hackett, S. F., Singer, J. H., and Campochiaro, P. A. (1990) Growth factor responsiveness of human retinal pigment epithelial cells. *Invest. Ophthalmol. Vis. Sci.,* **31,** 839–846.

Letizia, C., Iannaccone, A., Cerci, S., Santi, G., Cilli, M., Coassin, S., Pannarale, M. R., Scavo, D., and Iannacone, A. (1997) Circulating endothelin-1 in non-insulin-dependent diabetic patients with retinopathy. *Horm. Metab. Res.,* **29,** 247–251.

Levin, E. R. (1995) Endothelins. *N. Engl. J. Med.,* **333,** 356–363.

Lowe, W. L., Jr., Florkiewicz, R. Z., Yorek, M. A., Spanheimer, R. G., and Albrecht, B. N. (1995) Regulation of growth factor mRNA levels in the eyes of diabetic rats. *Metabolism,* **44,** 1038.

Lu, M., and Adamis, A. P. (2002) Vascular endothelial growth factor gene regulation and action in diabetic retinopathy. *Ophthalmol. Clin. North. Am.,* **15,** 69–79.

Lui, V. W., and Grandis, J. R. (2002). EGFR-mediated cell cycle regulation. *Anticancer. Res.,* **22,** 1–11

Lynch, J. J., Ferro, T. J., Blumenstock, F. A., Brockenauer, A. M., and Malik, A. B. (1990) Increased endothelial albumin permeability mediated by protein kinase C activation. *J. Clin. Invest.,* **85,** 991–998.

Madge, L. A., and Pober, J. S. (2001) Signaling in vascular endothelial cells. *Exp. Mol. Pathol.,* **70,** 317–325.

Mark, K. S., and Miller, D. W. (1999) Increased permeability of primary cultured brain microvessel endothelial cell monolayers following TNF-alpha exposure. *Life Sci.,* **64,** 1941–1953.

Marsh, S., Nakhoul, F. M., Skorecki, K., Rubin, A., Miller, B. P., Leibu, R., Levy, N. S., and Levy, A. P. (2000) Hypoxic induction of vascular endothelial growth factor is markedly decreased in diabetic individuals who do not develop retinopathy. *Diabetes Care,* **23,** 1375–1380.

Marx, M., Perlmutter, R. A., and Madri, J. A. (1994) Modulation of platelet-derived growth factor receptor expression in microvascular endothelial cells during in vitro angiogenesis. *J. Clin. Invest.,* **93,** 131–139.

Mathews, M. K., Merges, C., McLeod, D. S., and Lutty, G. A. (1997) Vascular endothelial growth factor and vascular permeability changes in human diabetic retinopathy. *Invest. Ophthalmol. Vis. Sci.,* **38,** 2729–2741.

Matsuda, H., Koyama, H., Sato, H., Sawada, J., Itakura, A., Tanaka, A., Matsumoto, M., Konno, K., Ushio, H., and Matsuda, K. (1998) Role of nerve growth factor in cutaneous wound healing: accelerating effects in normal and healing-impaired diabetic mice. *J. Exp. Med.,* **187,** 297–306.

Merimee, T. J., Zapf, J., and Froesch, E. R. (1983) Insulin-like growth factors. Studies in diabetics with and without retinopathy. *N. Engl. J. Med.,* **309,** 527–530.

Merwin, J. R., Newman, W., Beall, L. D., Tucker, A., and Madri, J. (1991) Vascular cells respond differentially to transforming growth factors beta 1 and beta 2 in vitro. *Am. J. Pathol.,* **138,** 37–51.

Meyer-Schwickerath, R., Pfeiffer, A., Blum, W. F., Freyberger, H., Klein, M., Losche, C., Rollmann, R., and Schatz, H. (1993) Vitreous levels of the insulin-like growth factors I and II, and the insulin-like growth factor binding proteins 2 and 3, increase in neovascular eye disease. Studies in nondiabetic and diabetic subjects. *J. Clin. Invest.,* **92,** 2620–2625.

Miller, J. W., Adamis, A. P., and Aiello, L. P. (1997) Vascular endothelial growth factor in ocular neovascularization and proliferative diabetic retinopathy. *Diabetes Metab. Rev.,* **13,** 37–50.

Mitamura, Y., Tashimo, A., Nakamura, Y., Tagawa, H., Ohtsuka, K., Mizue, Y., and Nishihira, J. (2002) Vitreous levels of placenta growth factor and vascular endothelial growth factor in patients with proliferative diabetic retinopathy. *Diabetes Care,* **25,** 2352.

Morbidelli, L., Orlando, C., Maggi, C. A., Ledda, F., and Ziche, M. (1995) Proliferation and migration of endothelial cells is prompted by endothelins via activation of ETB receptors. *Am. J. Physiol.,* **269,** H686–H695.

Mori, K., Duh, E., Gehlbach, P., Ando, A., Takahashi, K., Pearlman, J., Mori, K., Yang, H. S, Zack, D. J, Ettyreddy D., Brough D. E., Wei, L. L., and Campochiaro, P. A. (2001) Pigment epithelium-derived factor inhibits retinal and choroidal neovascularization. *J. Cell Physiol.,* **188,** 253–263.

Morise, T., Takeuchi, Y., Kawano, M., Koni, I., and Takeda, R. (1995) Increased plasma levels of immunoreactive endothelin and von Willebrand factor in NIDDM patients. *Diabetes Care,* **18,** 87–89.

Muller, G., Behrens, J., Nussbaumer, U., Bohlen, P., and Birchmeier, W. (1987) Inhibitory action of transforming growth factor beta on endothelial cells. *Proc. Natl. Acad. Sci. U. S. A.,* **84,** 5600–5604.

Muthukrishnan, L., Warder, E., and McNeil, P. L. (1991) Basic fibroblast growth factor is efficiently released from a cytolsolic storage site through plasma membrane disruptions of endothelial cells. *J. Cell Physiol,* **148,** 1–16.

Nakamura, T., Nawa, K., and Ichihara, A. (1984) Partial purification and characterization of hepatocyte growth factor from serum of hepatectomized rats. *Biochem. Biophys. Res. Commun.,* **122,** 1450–1459.

Nakamura, Y., Morishita, R., Higaki, J., Kida, I., Aoki, M., Moriguchi, A., Yamada, K., Hayashi, S., Yo, Y., Nakano, H., Matsumoto, K., Nakamura, T., and Ogihara, T. (1996) Hepatocyte growth factor is a novel member of the endothelium-specific growth factors: Additive stimulatory effect of hepatocyte growth factor with basic fibroblast growth factor but not with vascular endothelial growth factor. *J. Hypertens.,* **14,** 1967–1972.

Nakao-Hayashi, J., Ito, H., Kanayasu, T., Morita, I., and Murota, S. (1992) Stimulatory effects of insulin and insulin-like growth factor I on migration and tube formation by vascular endothelial cells. *Atherosclerosis,* **92,** 141–149.

Neely, K. A., Quillen, D. A., Schachat, A. P., Gardner, T. W., and Blankenship, G. W. (1998) Diabetic retinopathy. *Med. Clin. North. Am.,* **82,** 847–876.

Nezu, E., Ohashi, Y., Kinoshita, S., and Manabe, R. (1992) Recombinant human epidermal growth factor and corneal neovascularization. *Jpn. J. Ophthalmol.,* **36,** 401–406.

Nicosia, R. F., Nicosia, S. V., and Smith, M. (1994) Vascular endothelial growth factor, platelet-derived growth factor, and insulin-like growth factor-1 promote rat aortic angiogenesis in vitro. *Am. J. Pathol.,* **145,** 1023–1029.

Nishimura, M., Ikeda, T., Ushiyama, M., Kinoshita, S., and Yoshimura, M. (2000) Changes in vitreous concentrations of human hepatocyte growth factor (hHGF) in proliferative diabetic retinopathy: Implications for intraocular hHGF production. *Clin. Sci.,* **98,** 9–14.

Nishimura, M., Ikeda, T., Ushiyama, M., Nanbu, A., Kinoshita, S., and Yoshimura, M. (1999) Increased vitreous concentrations of human hepatocyte growth factor in prolilferative diabetic retinopathy. *J. Clin. Endocrinol. Metab.,* **84,** 659–662.

Noma, H., Funatsu, H., Yamashita. H., Kitano, S., Mishima, H. K., and Hori, S. (2002) Regulation of angiogenesis in diabetic retinopathy: Possible balance between vascular endothelial growth factor and endostatin. *Arch. Ophthalmol.,* **120,** 1075–1080.

Ogata, N., Nishikawa, M., Nishimura, T., Mitsuma, Y., and Matsumura, M. (2002) Unbalanced vitreous levels of pigment epithelium-derived factor and vascular endothelial growth factor in diabetic retinopathy. *Am. J. Ophthalmol.,* **134,** 348–353.

Ogata, N., Tombran-Tink, J., Nishikawa, M., Nishimura, T., Mitsuma, Y., Sakamoto, T., and Matsumura, M. (2001) Pigment epithelium-derived factor in the vitreous is low in diabetic retinopathy and high in rhegmatogenous retinal detachment. *Am. J. Ophthalmol.,* **132,** 378–382.

Oh, H., Takagi, H., Suzuma, K., Otani, A., Matsumura, M., and Honda, Y. (1999) Hypoxia and vascular endothelial growth factor selectively up-regulate angiopoietin-2 in bovine microvascular endothelial cells. *J. Biol. Chem.,* **274,** 15732–15739.

Ohno-Matsui, K., Hirose, A., Yamamoto, S., Saikia, J., Okamoto, N., Gehlbach, P., Duh, E. J., Hackett, S., Chang, M., Bok, D., Zack, D. J., and Campochiaro, P. A. (2002) Inducible expression of vascular endothelial growth factor in adult mice causes severe proliferative retinopathy and retinal detachment. *Am. J. Pathol.,* **160,** 711–719.

Oku, H., Kida, T., Sugiyama, T., Hamada, J., Sato, B., and Ikeda, T. (2001) Possible involvement of endothelin-1 and nitric oxide in the pathogenesis of proliferative diabetic retinopathy. *Retina,* **21,** 674–651.

Okumura, K., Nishiura, T., Awaji, Y., Kondo, J., Hashimoto, H., and Ito, T. (1991) 1,2-diacylglycerol content and its composition in thoracic aorta of diabetic rats. *Diabetes,* **40,** 820–824.

Ozaki, H., Seo, M. S., Ozaki, K., Yamada, H., Yamada, E., Okamoto, N., Hofmann, F., Wood, J. M., and Campochiaro, P. A. (2000) Blockade of vascular endothelial cell growth factor receptor signaling is sufficient to completely prevent retinal neovascularization. *Am. J. Pathol.,* **156,** 697–707.

Pascal, M. M., Forrester, J. V., and Knott, R. M. (1999) Glucose-mediated regulation of transforming growth factor-beta (TGF-$\beta$) and TGF-$\beta$ receptors in human retinal endothelial cells. *Curr. Eye Res.,* **19,** 162–170.

Patel, B., Hiscott, P., Charteris, D., Mather, J., McLeod, D., and Boulton, M. (1994) Retinal and preretinal localisation of epidermal growth factor, transforming growth factor alpha, and their receptor in proliferative diabetic retinopathy. *Br. J. Ophthalmol.,* **78,** 714–718.

Patz, A. (1982) Clinical and experimental studies on retinal neovascularization. XXXIX Edward Jackson Memorial Lecture. *Am. J. Ophthalmol.,* **94,** 715–743.

Pe'er, J., Folberg, R., Itin, A., Gnessin, H., Hemo, I., and Keshet, E. (1996) Upregulated expression of vascular endothelial growth factor in proliferative diabetic retinopathy. *Br. J. Ophthalmol.,* **80,** 241–245.

Pertovaara, L., Kaipainen, A., Mustonen, T., Orpana, A., Ferrara, N., Saksela, O., and Alitalo, K. (1994) Vascular endothelial growth factor is induced in response to transforming growth factor-beta in fibroblastic and endothelial cells. *J. Biol. Chem.,* **269,** 6271–6274.

Pfeiffer, A., Spranger, J., Meyer-Schwickerath R., and Schatz, H. (1997) Growth factor alterations in advanced diabetic retinopathy: A possible role of blood retina barrier breakdown. *Diabetes,* **46,** S26.

Phillips, G. D., Whitehead, R. A., Stone, A. M., Ruebel, M. W., Goodkin, M. L., and Knighton, D. R. (1993) Transforming growth factor beta (TGF-B) stimulation of angiogenesis: an electron microscopic study. *J. Submicrosc. Cytol. Pathol.,* **25,** 149–155.

Pober, J. S. (1998) Activation and injury of endothelial cells by cytokines. *Pathol. Biol.,* **46,** 159–163.

Pober, J. S., and Cotran, R. S. (1990) Cytokines and endothelial cell biology. *Physiol. Rev.,* **70,** 427–451.

Poulsen, J. D. (1953) Diabetes and anterior pituitary deficiency. *Diabetes,* **2,** 7–12.

Prestrelski, S. J., Fox, G. M., and Arakawa, T. (1992) Binding of heparin to basic fibroblast growth factor induces a conformational change. *Arch. Biochem. Biophys.,* **293,** 314–319.

Pugliese, G., Tilton, R. G., and Williamson, J. R. (1991) Glucose-induced metabolic imbalances in the pathogenesis of diabetic vascular disease. *Diabetes Metab. Rev.,* **7,** 35–59.

Punglia, R. S., Lu, M., Hsu, J., Kuroki, M., Tolentino, M. J., Keough. K., Levy, A. P., Levy, N. S., Goldberg, M. A., D'Amato, R. J., and Adamis, A. P. (1997) Regulation of vascular endothelial growth factor expression by insulin-like growth factor I. *Diabetes,* **46,** 1619–1026.

Qi, J. H., and Claesson-Welsh, L. (2001) VEGF-induced activation of phosphoinositide 3-kinase is dependent on focal adhesion kinase. *Exp. Cell Res.,* **263,** 173–182.

Raines, E. W. (1993) In: *Biology of Platelet-Derived Growth Factor*, Edited by Westermark, B., and Sorg, C., pp. 1–114. Basel, Switzerland, Karger.

Raines, E. W., Brown-Pope, D. F., and Ross, R. (1990) In: *Platelet-Derived Growth Factor*, Edited by Sporn, M. B., and Roberts, A. B., pp. 173–262. Heidelberg, Springer-Verlag.

Raychaudhuri, S. K., Raychaudhuri, S. P., Weltman, H., and Farber, E. M. (2001) Effect of nerve growth factor on endothelial cell biology: Proliferation and adherence molecule expression on human dermal microvascular endothelial cells. *Arch. Dermatol. Res.,* **293,** 291–295.

Risau, W., Drexler, H., Mironov, V., Smits, A., Siegbahn, A., Funa, K., and Heldin, C. H. (1992) Platelet-derived growth factor is angiogenic in vivo. *Growth Factors,* **7,** 261–266.

Roberts, A. B., Sporn, M. B., Assoian, R. K., Smith, J. M., Roche, N. S., Wakefield, L. M., Heine, U. I., Liotta, L. A., Falanga, V., Kehrl, J. H., and Fauci, A. S. (1986) Transforming growth factor type beta: rapid induction of fibrosis and angiogenesis in vivo and stimulation of collagen formation in vitro. *Proc. Natl. Acad. Sci. U. S. A.,* **83,** 4167–4171.

Robinson, C. J., and Stringer, S. E. (2001) The splice variants of vascular endothelial growth factor (VEGF) and their receptors. *J. Cell Sci.,* **114,** 853–865.

Robinson, G. S., Pierce, E. A., Rook, S. L., Foley, E., Webb, R., and Smith, L. E. (1996) Oligodeoxynucleotides inhibit retinal neovascularization in a murine model of proliferative retinopathy. *Proc. Natl. Acad. Sci. U. S. A.,* **93,** 4851–4856.

Rubanyi, G. M., and Polokof, M. A. (1994) Endothelins: Molecular biology, biochemistry, pharmacology, physiology and pathophysiology. *Pharmacol. Rev.,* **46,** 326–414.

Ruscetti, F. W., Birchenal-Roberts, M. C., McPherson, J. M., and Wiltrout. R. H. (1998) Transforming growth factor $\beta1$. In: *Cytokines,* Edited by Mire-Luis A., and Thorpe R., pp. 415–432. San Diego, Academic Press.

Sakamoto, T., Ueno, H., Sonoda, K., Hisatomi, T., Shimizu, K., Ohashi, H., and Inomata, H. (2000) Blockade of TGF-beta by in vivo gene transfer of a soluble TGF-beta type II receptor in the muscle inhibits corneal opacification, edema and angiogenesis. *Gene Ther.,* **7,** 1915–1924.

Saksela, O., Moscatelli, D., Sommer, A., and Rifkin, D, B. (1988) Endothelial cell-derived heparan sulfate binds basic fibroblast growth factor and protects it from proteolytic degradation. *J. Cell Biol.,* **107,** 743–751.

Sakurai, T., Yanagisawa, M., and Masaki, T. (1992) Molecular characterization of endothelin receptors. *Trends Pharmacol. Sci.,* **13,** 103–108.

Salani, D., Taraboletti, G., Rosano, L., Di Castro, V., Borsotti, P., Giavazzi, R., and Bagnato, A. (2000) Endothelin-1 induces an angiogenic phenotype in cultured endothelial cells and stimulates neovascularization in vivo. *Am. J. Pathol.,* **157,** 1703–1711.

Sato, N., Beitz, J. G., Kato, J., Yamamoto, M., Clark J. W., Calabresi, P., Raymond, A., and Frackelton, A. R., Jr. (1993) Platelet-derived growth factor indirectly stimulates angiogenesis in vitro. *Am. J. Pathol.,* **142,** 1119–1130.

Schweigerer, L., Ferrara, N., Haaparanta, T., Neufeld, G., and Gospodarowicz, D. (1988) Basic fibroblast growth factor: Expression in cultured cells derived from corneal endothelium and lens epithelium. *Exp. Eye Res.,* **46,** 71–80.

Schweigerer, L., Neufeld, G., Friedman, J., Abraham, J. A., Fiddes, J. C., and Gospodarowicz, D. (1987) Capillary endothelial cells express basic fibroblast growth factor, a mitogen that promotes their own growth. *Nature,* **325,** 257–259.

Seymour, L. W., Shoaibi, M. A., Martin, A., Ahmed, A., Elvin, P., Kerr, D. J., and Wakelam, M. J. (1996) Vascular endothelial growth factor stimulates protein kinase C-dependent phospholipase D activity in endothelial cells. *Lab. Invest.,* **75,** 427–437.

Sheetz, M. J., and King, G. L. (2002) Molecular understanding of hyperglycemia's adverse effects for diabetic complications. *JAMA,* **288,** 2579–2588.

Shimizu, K., Kobayashi, Y., and Muraoka, K. (1981) Midperipheral fundus involvement in diabetic retinopathy. *Ophthalmology,* **88,** 601–612.

Shinoda, K., Ishida, S., Kawashima, S., Wakabayashi, T., Matsuzaki, T., Takayama, M., Shinmura, K., and Yamada, M. (1999) Comparison of the levels of hepatocyte growth factor and vascular endothelial growth factor in aqueous fluid and serum with grades of retinopathy in patients with diabetes mellitus. *Br. J. Ophthalmol.,* **83,** 834–837.

Sholley, M. M., Ferguson, G. P., Seibel, H. R., Montour, J. L., and Wilson, J. D. (1984) Mechanisms of neovascularization. Vascular sprouting can occur without proliferation of endothelial cells. *Lab. Invest.,* **51,** 624–634.

Simo, R., Lecube, A., Segura, R. M., Garcia, Arumi, J., and Hernandez, C. (2002) Free insulin growth factor-I and vascular endothelial growth factor in the vitreous fluid of patients with proliferative diabetic retinopathy. *Am. J. Ophthalmol.,* **134,** 376–382.

Singh, V. K., Chader, G. J., and Rodriguez, I. R. (1998) Structural and comparative analysis of mouse gene for pigment epithelium-derived factor (PEDF). *Mol. Vis.,* **4,** 7.

Sivalingam, A., Kenney, J., Brown, G. C., Benson, W. E., and Donoso, L. (1990) Basic fibroblast growth factor levels in the vitreous of patients with proliferative diabetic retinopathy. *Arch. Ophthalmol.,* **108,** 869–872.

Smits, A., Hermanson, M., Nester, M., Karnushina, I., Heldin, C. H., Westermark, B., and Funa, K. (1989) Rat brain capillary endothelial cells express functional PDGF B-type receptors. *Growth Factors,* **2,** 1–8.

Sommer, A., and Rifkin, D. B. (1989) Interaction of heparin with human basic fibroblast growth factor: Protection of the angiogenic protein from proteolytic degradation by a glycosaminoglycan. *J. Cell Physiol.,* **138,** 215–220.

Sporn, M. B., and Roberts, A. B. (1988) Peptide growth factors are multifunctional. *Nature,* **332,** 217–219.

Spranger, J., Meyer-Schwickerath, R., Klein, M., Schatz, H., and Pfeiffer, A. (1995) TNF-alpha level in the vitreous body. Increase in neovascular eye diseases and proliferative diabetic retinopathy. *Med. Klin.,* **90,** 134–137.

Spranger, J., Osterhoff, M., Reimann, M., Mohlig, M., Ristow, M., Francis, M. K., Cristofalo, V., Hammes, H. P., Smith, G., Boulton, M., and Pfeiffer, A. F. (2001) Loss of the antiangiogenic pigment epithelium-derived factor in patients with angiogenic eye disease. *Diabetes,* **50,** 2641–2645.

Stanimirovic, D. B., Bertrand, N., McCarron, R., Uematsu, S., and Spatz, M. (1994) Arachidonic acid release and permeability changes induced by endothelins in human cerebromicrovascular endothelium. *Acta Neurochir. Suppl.,* **60,** 71–75.

Stavri, G. T., Hong, Y., Zachary, IC., Breier, G., Baskerville, P. A., Yla-Herttuala, S., Risau, W., Martin, J. F., and Erusalimsky, J. D. (1995) Hypoxia and platelet-derived growth factor synergistically upregulate the expression of vascular endothelial growth factor in vascular smooth muscle cells. *FEBS Lett.,* **358,** 311–315.

Stavri, G. T., Zachary, I. C., Baskerville, P. A., Martin, J. F., and Erusalimsky, J. D. (1995) Basic fibroblast growth factor upregulates the expression of vascular endothelial growth factor in vascular smooth muscle cells. Synergistic interaction with hypoxia. *Circulation,* **92,** 11–14.

Stellmach, V., Crawford, S. E., Zhou, W., and Bouck, N. (2001) Prevention of ischemia–induced retinopathy by the natural ocular antiangiogenic agent pigment epithelium-derived factor. *Proc. Natl. Acad. Sci. U. S. A.,* **98,** 2593–2597.

Sternfeld, M. D., Robertson, J. E., Shipley, G. D., Tsai, J., and Rosenbaum, J. T. (1989) Cultured human retinal pigment epithelial cells express basic fibroblast growth factor and its receptor. *Curr. Eye Res.,* **8,** 1029–1037.

Sumner, M. J., Cannon, T. R., Mundin, J. W., White, D. G., and Watts, I. S. (1992) Endothelin ETA and ETB receptors mediate vascular smooth muscle contraction. *Br. J. Pharmacol.,* **107,** 858–860.

Suzuma, K., Naruse, K., Suzuma, I., Takahara, N., Ueki, K., Aiello, L. P., and King, G. L. (2000) Vascular endothelial growth factor induces expression of connective tissue growth factor via KDR, Flt1, and phosphatidylinositol 3-kinase-akt-dependent pathways in retinal vascular cells. *J. Biol. Chem.,* **275,** 40725–40731.

Takagi, Y., Kashiwagi, A., Tanaka, Y., Asahian, T., Kikkawa, R., and Shigeta, Y. (1995) Significance of fructose-induced protein oxidation and formation of advanced glycation end product. *J. Diabetes Complications,* **9,** 89–91.

Takahashi, T., and Shibuya, M. (1997) The 230 kDa mature form of KDR/Flk-1 (VEGF receptor-2) activates the PLC-gamma pathway and partially induces mitogenic signals in NIH3T3 fibroblasts. *Oncogene,* **14,** 2079–2089.

Takahashi, K., Ghatei, M. A., Lam, H. C., O'Halloran, D. J., and Bloom, S. R. (1990) Elevated plasma endothelin in patients with diabetes mellitus. *Diabetologia,* **33,** 306–310.

Tanaka, Y., Katoh, S., Hori, S., Miura, M., and Yamashita, H. (1997) Vascular endothelial growth factor in diabetic retinopathy. *Lancet,* **349,** 1520.

Teicher, B. A., Alvarez, E., Menon, K., Esterman, M. A., Considine, E., Shih, C., and Faul, M. M. (2002) Antiangiogenic effects of a protein kinase C beta-selective small molecule. *Cancer Chemother, Pharmacol.,* **49,** 69–77.

Terman, B. I., Dougher-Vermazen, M., Carrion, M. E., Dimitrov, D., Armellino, D. C., Gospodarowicz, D., and Bohlen, P. (1992) Identification of the KDR tyrosine kinase as a receptor for vascular endothelial growth factor. *Biochem. Biophys. Res. Commun.,* **187,** 1597–1586.

Thrailkill, K. M., Quattrin, T., Baker, L., Kuntze, J. E., Compton, P. G., and Martha, P. M., Jr. (1999) Cotherapy with recombinant human insulin-like growth factor I and insulin improves glycemic control in type 1 diabetes. RhIGF-I in IDDM Study Group. *Diabetes Care,* **22,** 585–592.

Vigne, P., Marsault, R., Breittmayer, J. P., and Frelin, C. (1990) Endothelin stimulates phosphoinositol hydrolysis and DNA synthesis in brain capillary endothelial cells. *Biochem. J.,* **266,** 415–420.

Vinals, F., and Pouyssegur, J. (2001) Transforming growth factor beta1 (TGF-beta1) promotes endothelial cell survival during in vitro angiogenesis via an autocrine emchanism implicating TGF-alpha signaling. *Mol. Cell Biol.,* **21,** 7218–7230.

Vlassara, H. (1997) Recent progress in advanced glycation end products and diabetic complications. *Diabetes,* **46,** S19.

Vlassara, H. (2001) The AGE-receptor in the pathogenesis of diabetic complications. *Diabetes Metab. Res. Rev.,* **17,** 436–443.

Wang, Q., Dills, D. G., Klein, R., Klein, B. E., and Moss, S. E. (1995) Does insulin-like growth factor I predict incidence and progression of diabetic retinopathy? *Diabetes,* **44,** 161–164.

Wellner, M., Maasch, C., Kupprion, C., Lindschau, C., Luft, F. C., and Haller, H. (1999) The proliferative effect of vascular endothelial growth factor requires protein kinase C-alpha and protein kinase C-zeta. *Arterioscler. Thromb. Vasc. Biol.,* **19,** 178–185.

Williams, B., Gallacher, B., Patel, H., and Orme, C. (1997) Glucose-induced protein kinase C activation regulates vascular permeability factor mRNA expression and peptide production by human vascular smooth muscle cells in vitro. *Diabetes,* **46,** 1497–1503.

Williamson, J. R., Chang, K., Frangos, M., Hasan, K. S., Ido, Y., Kawamura, T., Nyengaard, J. R., van, den, Enden, M., Kilo, C., and Tilton, R. G. (1993) Perspectives in diabetes. Hyperglycemic pseudohypoxia and diabetic complications. *Diabetes,* **42,** 801–813.

Williamson, J. R., and Kilo, C. (1983) Capillary basement membrane in diabetes. *Diabetes,* **32,** 96–100.

Wong, C. G., Rich, K. A., Liaw, L. H., Hsu, H. T., and Berns, M. W. (2001) Intravitreal VEGF and bFGF produce florid retinal neovascularization and hemorrhage in the rabbit. *Curr. Eye Res.,* **22,** 140–147.

Woost, P. G., Jumblatt, M. M., Eiferman, R. A., and Schultz, G. S. (1992) Growth factors and corneal endothelial cells: II. Characterization of epidermal growth factor receptor from bovine corneal endothelial cells. *Cornea,* **11,** 11–19.

Wu, Y. Q., Notario, V., Chader, G. J., and Becerra, S. P. (1995) Identification of pigment epithelium-derived factor in the interphotoreceptor matrix of bovine eyes. *Prot. Exp. Purif.,* **6,** 447–456.

Xia, P., Inoguchi, T., Kern, K. S., Engerman, R. L., Oats, P. J., and King, G. L. (1994) Characterization of the mechanism for the chronic activation of diacylglycerol-protein kinase C in diabetes and hypergalactosaemia. *Diabetes,* **43,** 1122–1129.

Yanagisawa, M., Kurihara, H., Kimura, S., Tomobe, Y., Kobayashi, M., Mitsui, Y., Yazaki, Y., Goto, K., and Masaki, T. (1988) A novel potent vasoconstrictor peptide produced by vascular endothelial cells. *Nature,* **332,** 411–415.

Yang, E. Y., and Moses, H. L. (1990) Transforming growth factor beta 1-induced changes in cell migration, proliferation, and angiogenesis in the chicken chorioallantoic membrane. *J. Cell Biol.,* **111,** 731–741.

Yokota, T., Ma, R. C., Park, J. Y., Isshiki, K., Sotiropoulos, K. B., Rauniyar, R. K., Bornfeldt, K. E., and King, G. L. (2003) Role of protein kinase C on the expression of platelet-derived growth factor and endothelin-1 in the retina of diabetic rats and cultured retinal capillary pericytes. *Diabetes,* **52,** 838–845.

Yuuki, T., Kanda, T., Kimura, Y., Kotajima, N., Tamura, J., Kobayashi, I., and Kishi, S. (2001) Inflammatory cytokines in vitreous fluid and serum of patients with diabetic vitreoretinopathy. *J. Diabetes Complications,* **15,** 257–259.

Zhou, Z., Yang, X. M., Xie. Y. Z, and Yin, Z. Y. (2002) Vascular endothelial growth factor gene expression regulated by protein kinase C pathway in endothelial cells during hypoxia. *Space Med. Med. Eng.,* **15,** 322–326.

